# Flow cytometry analysis of *Clostridium beijerinckii* NRRL B-598 populations exhibiting different phenotypes induced by changes in cultivation conditions

**DOI:** 10.1186/s13068-018-1096-x

**Published:** 2018-04-06

**Authors:** Barbora Branska, Zora Pechacova, Jan Kolek, Maryna Vasylkivska, Petra Patakova

**Affiliations:** Department of Biotechnology, University of Chemistry and Technology Prague, Technicka 5, 166 28 Prague, Czech Republic

**Keywords:** Cytometry, Clostridium, Fluorescence staining, Viability, ABE fermentation, Butanol, Sporulation, Stress

## Abstract

**Background:**

Biobutanol production by clostridia via the acetone–butanol–ethanol (ABE) pathway is a promising future technology in bioenergetics , but identifying key regulatory mechanisms for this pathway is essential in order to construct industrially relevant strains with high tolerance and productivity. We have applied flow cytometric analysis to *C. beijerinckii* NRRL B-598 and carried out comparative screening of physiological changes in terms of viability under different cultivation conditions to determine its dependence on particular stages of the life cycle and the concentration of butanol.

**Results:**

Dual staining by propidium iodide (PI) and carboxyfluorescein diacetate (CFDA) provided separation of cells into four subpopulations with different abilities to take up PI and cleave CFDA, reflecting different physiological states. The development of a staining pattern during ABE fermentation showed an apparent decline in viability, starting at the pH shift and onset of solventogenesis, although an appreciable proportion of cells continued to proliferate. This was observed for sporulating as well as non-sporulating phenotypes at low solvent concentrations, suggesting that the increase in percentage of inactive cells was not a result of solvent toxicity or a transition from vegetative to sporulating stages. Additionally, the sporulating phenotype was challenged with butanol and cultivation with a lower starting pH was performed; in both these experiments similar trends were obtained—viability declined after the pH breakpoint, independent of the actual butanol concentration in the medium. Production characteristics of both sporulating and non-sporulating phenotypes were comparable, showing that in *C. beijerinckii* NRRL B-598, solventogenesis was not conditional on sporulation.

**Conclusion:**

We have shown that the decline in *C. beijerinckii* NRRL B-598 culture viability during ABE fermentation was not only the result of accumulated toxic metabolites, but might also be associated with a special survival strategy triggered by pH change.

## Background

The production of butanol from clostridia through acetone–butanol–ethanol (ABE) fermentation is a well- established process with a long and rich history involving many industrial productions worldwide [1]. Moreover, butanol is a very promising biofuel, in many respects, better than ethanol, having a higher low heating value (LHV), higher energy content per volume, can be used in diesel engines and is less corrosive [2, 3]. Unfortunately, restoration of industrial scale butanol production is not yet in sight, despite continuing efforts to re-establish the industry (e.g. green biologics plans to produce butanol in the USA) [4] and interest in the technology persists in China [5]. Nowadays, fermentative production of butanol cannot compete economically with chemically synthesized butanol or first generation ethanol. The main bottlenecks in the process are low butanol yields and final concentration, which increase the final product cost. Most research in the field is therefore focused on overcoming these obstacles. Different strategies have been used including utilization of low value waste material as a substrate [6–10], by optimizing cultivation conditions and process design employing, e.g. simultaneous saccharification and fermentation (instead of separate hydrolysis and fermentation), continuous or semi-continuous cultivation, the application of immobilized cells [11–13], or use of symbiotic co-cultures [14, 15]. Other approaches include removal of butanol in situ from the medium [8, 16–18], adaptation [19, 20] or engineering of strains [21–25] for higher tolerance, production and improved yield by altering metabolite flux (e.g. by reduction of by-product formation via disruption of respective genes [26]), enhancement of expression of genes responsible for solvent formation [27] or by totally suppressing acid formation and directly stimulating butanol formation from saccharides [28]. The issue of tolerance and solvent titres seems to be a tricky one, as it might be assumed that the final butanol concentration is limited by the strain’s tolerance to butanol. However, many experiments have revealed that an improved ability of clostridia to tolerate/survive higher concentrations of butanol might not necessarily be commensurate with increased production [29, 30].

Through database searches of different species, we can identify strains with superior butanol tolerance, e.g. *Pseudomonas putida* (up to 50 g/L) [31], which suggests that there might exist powerful natural mechanisms to cope with butanol stress, providing us with an optimistic perspective that construction of highly tolerant producing strains is realistic. However, first, the main cause of limited production and a decline in viability during ABE fermentation must be properly understood. The second point is the one that we want to touch upon in this work. Generally, during their life cycle, solventogenic clostridia produce substances that are detrimental to them, including the production of acetic and butyric acids that decrease the pH, the formation of undissociated molecules that are able to penetrate the cell membrane and also cause a decrease in intracellular pH, and the inability of clostridia to maintain a transmembrane pH gradient [1, 32]. Solventogenesis, to some extent, attenuates the pH pressure by partial reutilization of acids in the production of solvents—substances provoking a range of stress responses that again threaten clostridia [33]. Moreover, solventogenesis is usually accompanied by sporulation and the formation of autolysins [34], enzymes contributing to the lysis of cell cultures. Nevertheless, all processes appear to be tightly regulated, well balanced and ensure culture survival. Transcriptomic, proteomic and metabolomics approaches have been used to identify cell responses to self-generated stressors. Even more information would be gained if such studies were supplemented with data on other cell features such as morphological status and the proportion of viable cells. Until recently, the overall ability of cells to survive was mostly measured as colony forming units (CFU) and changes in morphology were observed microscopically. Introducing high throughput methodologies such as flow cytometry (FC) into the analysis of solventogenic clostridia [35–37] has enabled a more detailed insight. The tendency of clostridia to behave in unpredictable ways was seen in first attempts to introduce fluorescence based viability assays by Tracy et al. [37] or Jones et al. [38], who observed the opposite staining pattern of Syto 9 and propidium iodide (PI) than was expected (the majority of cells in exponential phase were stained with PI instead of Syto 9, suggesting the prevalence of inactive cells). However, this staining pattern has been shown to be a supporting tool for the identification of different morphological states. Linhová et al. [39] tested a wide range of fluorescent viability probes, suggesting that PI—an indicator of membrane integrity, carboxyfluorescein diacetate (CFDA)—an indicator of intracellular enzymatic activity and bis-oxonol (BOX)—a potentiometric probe penetrating depolarised cells, would be the most convenient for particular clostridial species. In 2016 our lab introduced a one-step methodology for the evaluation of clostridial viability, together with spore enumeration [35].

In this paper we have improved the methodology in terms of higher precision of spore counting and have applied the modified methodology on systematic screening for changes in viability/staining patterns during ABE fermentation, using either sporulating or non-sporulating phenotypes, a butanol challenge and a decrease in initial pH. A combination of two fluorescent probes was used: CFDA (an uncharged, non-fluorescent substance that can freely diffuse into cells, where it is enzymatically cleaved into a fluorescent product) and PI, the most well-known viability indicator that can only penetrate cells with a compromised cytoplasmic membrane.

## Results

### ABE fermentation with a sporulating phenotype

**Fig. 1:**
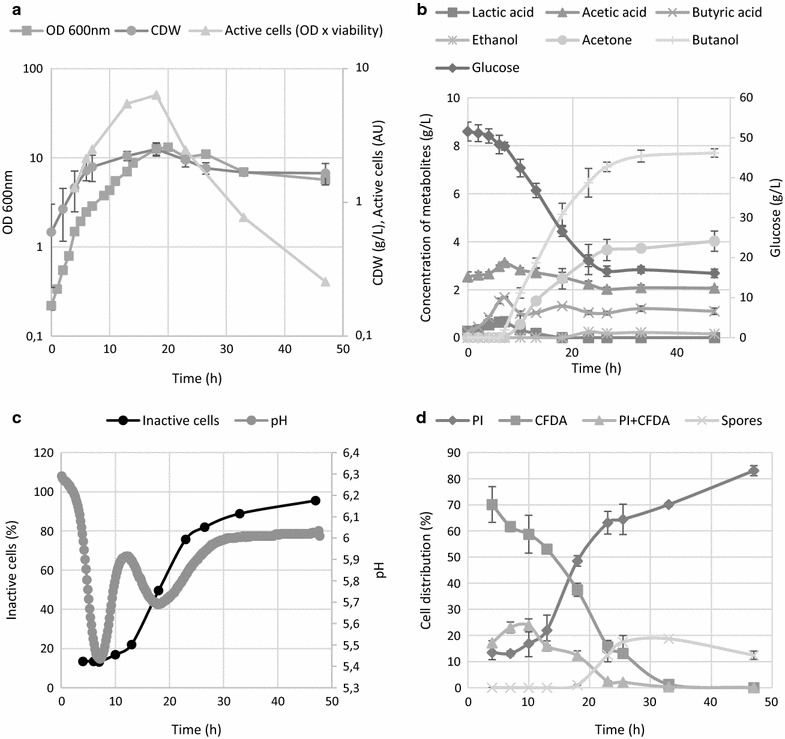
The time course of **a** cell growth and total amount of active cells (OD multiplied by reciprocal value of inactive cells from chart **c**), **b** metabolite formation, **c** pH and percentage of inactive cells, **d** distribution of different sub-populations according to their LS and fluorescence staining pattern for sporulation phenotype and ABE fermentation carried out on TYA medium without addition of external stress factors. Error bars represent standard deviations of three independent biological replicates, and calculated values are presented without error bars

*Clostridium beijerinckii* grown on TYA medium showed a typical bi-phasic ABE fermentation, starting with the formation of organic acids and then subsequent production of solvents accompanied by glucose consumption and partial reutilization of the organic acids (Fig. 1) took place. In comparison to the generally observed growth cessation after acidogenesis by *C. acetobutylicum* ATCC 824 [1, 25], the onset of solvent production in *C. beijerinckii* NRRL B-598 was associated with growth of a population, even though the growth rate slowed shortly before the culture pH reached its minimal value (Fig. 1).

**Fig. 2:**
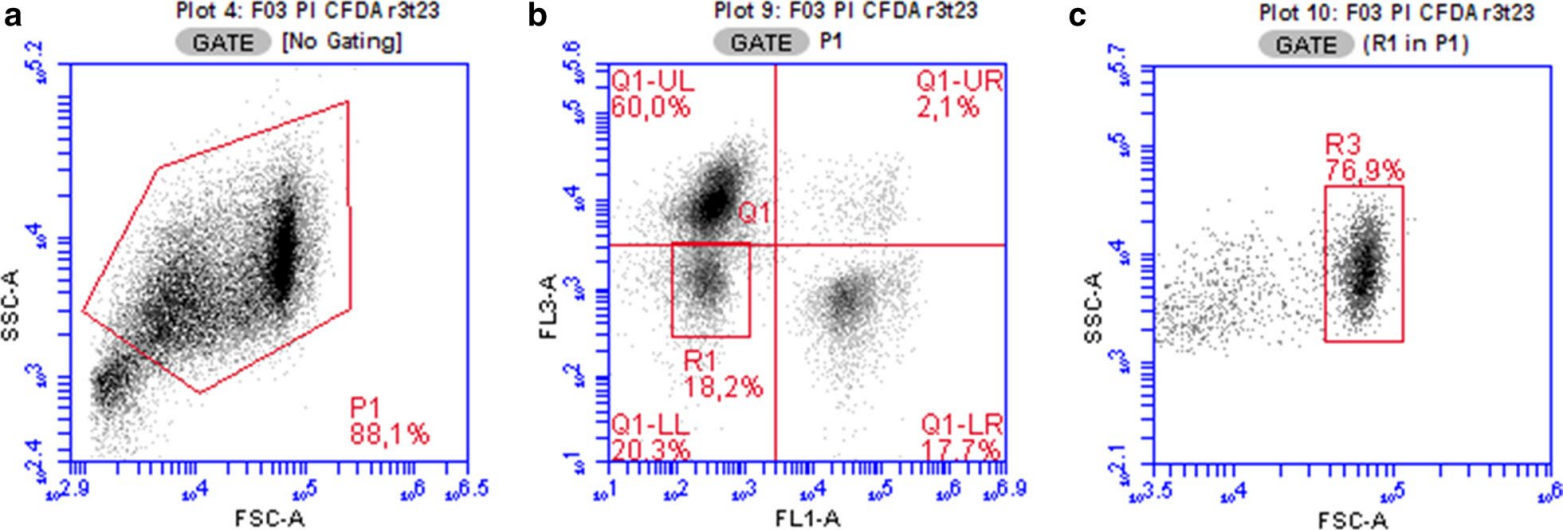
Identification of different sub-populations based on their light scatter and fluorescence signals after incubation with PI and CFDA. **a** Clostridial cells were separated from the background noise and gated as the P1 gate; only the P1 region was analysed for fluorescence, **b** fluorescence staining patterns: upper left (UL)—solely PI stained population, upper right (UR)—doubly stained cells, lower left (LL—non stained particles, R1—particles with fluorescence properties of mature spores released from mother cells, lower right (LR)—CFDA stained cells, and **c** identification of spores based on light scatter parameters (only particles occurring in the R1 gate were analysed in this step). *FL1* green fluorescence, *FL3* red fluorescence, *FSC* forward scatter signal, *SSC* side scatter signal

Viability and spore formation were measured using light scatter (LS) parameters of cells and spores together with staining with a combination of two fluorescent probes, carboxyfluorescein diacetate (CFDA) and propidium iodide (PI). The CFDA and PI mixed used in viability studies is a common approach [40] providing complex information about enzyme activity and membrane integrity of cells. Propidium iodide bears two positive charges that prevent diffusion into cells with retained membrane integrity [41], and if some dye is taken up by cells it should be immediately removed by active pumping. Once inside the cells it binds to nucleic acids and after binding, fluorescence is considerably multiplied enabling recognition of such stained cells by bright red fluorescence. By contrast, CFDA is a non-fluorescent electroneutral ester of fluorescein that can penetrate cell membranes by diffusion and, once inside cells, it is cleaved by nonspecific esterases to the carboxyfluorescein anion that cannot leak out by diffusion and accumulates within the cell providing green fluorescence. During ABE fermentation, at least 4 subpopulations could be recognized after CFDA and PI staining (Fig. 2)—(i) a population stained solely by PI (cells with damaged membranes with no or negligible enzyme activity or no ability to retain released fluorescein), (ii) a group of cells stained solely by CFDA (cells with intact membrane functions as well as enzyme activities), (iii) cells stained by both probes (cells with somehow weakened membrane functions but still with considerable enzyme activity and membrane integrity, at least to prevent fluorescein leakage; cell doublets; sporulating cells) and (iv) a non-stained population with LS parameters of a clostridial culture. This last group contained spores, non-cell particles and cell residues. Spores were subsequently recognised from the rest of unstained particles based on their typical LS signal (see Fig. 2).

**Fig. 3:**
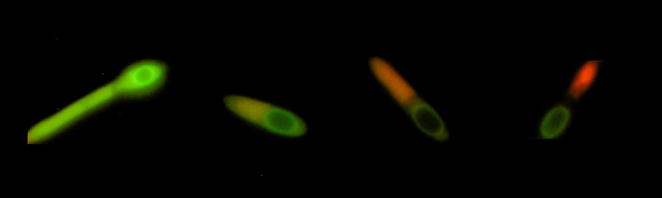
Development of staining pattern of *C. beijerinckii* NRRL B-598 during sporulation for cells stained by CFDA and PI (particular states were used from different microphotographs)

Active (viable) populations were identified in two groups, solely CFDA stained cells and combined PI and CFDA positive particles. In the case of a doubly stained pattern, it is questionable how to classify this population in terms of its viability/reproducibility. Doubly stained populations are usually formed by more cell types as described above, namely cell doublets, which, after cell sorting, form colonies on agar plates [42], double stained single cells in a particular physiological state [38] with lowered ability to efflux PI, and spore forming cells [35]. The development of fluorescence staining during sporulation is shown in Fig. 3—the mother cell appearing at the beginning as an active healthy cell gradually attenuates enzyme activity and loses the ability to prevent PI penetration, providing a change of microscopic view from bright green via orange to a clearly red cell. After the spore envelope is developed and matured, none of the probe can penetrate it and the spore remains non-fluorescent (although some intrinsic autofluorescence is present).

Despite the disputable culturability of doubly stained cells, in this work, they were considered as a viable/active population able to form acids or solvents. On the other hand, spores, together with solely PI positive cells, were counted as metabolically inactive populations. Although Shi et al. [43] questioned the reliability of PI as a viability indicator, due to its ability to penetrate cell membranes of living cells at particular stages of the life cycle, this seemed not to be the case with *C. beijerinckii* [35].

From the viability staining profile it is apparent that the number of active cells was highest until the metabolic shift, which occurred when the minimum pH was reached (Fig. 1c). A detailed analysis of the staining profile (Fig. 1d) shows that the proportion of cells solely stained by CFDA declined after the first measured point at the 4th hour. This was accompanied by an increase in the doubly stained population formed at this stage, mostly by incompletely separated cells forming short chains, so the percentage of the active population remained constantly high until the onset of solventogenesis and cell thickening (accumulation of granulose prior to sporulation). From the 10th hour, it is apparent that viability of the whole culture gradually decreased, even though glucose was present in excess and the butanol concentration was still low enough to not be a cause of cell damage and death. This phenomenon of losing viability was attributed to the start of sporulation and related to processes such as production of autolysins, scarifying of vegetative cells, and survival of a population in the form of spores. The total number of active cells (calculated as OD_600_ multiplied by the proportion of the active population) peaked around the 18th hour when the second pH minimum was reached and the butanol concentration was 5.1 g/L. Flow cytometry-determined viability was only 50% at this point. The influence of this sub-lethal concentration was tested in an additional experiment when 5 g/L of butanol was added to the cultivation medium prior to inoculation and cultivation was carried out under the same conditions as the reference cultivation with the sporulating phenotype. Moreover, to obtain a better insight into a possible role of sporulation in the decline in viability, an experiment under conditions for which it was experimentally proved that cells of *C. beijerinckii* NRRL B-598 do not sporulate, was performed. Non-sporulating cultures of *C. beijerinckii* NRRL B-598 were prepared on modified RCM medium [35, 44].

### ABE fermentation with non-sporulating phenotype

**Fig. 4:**
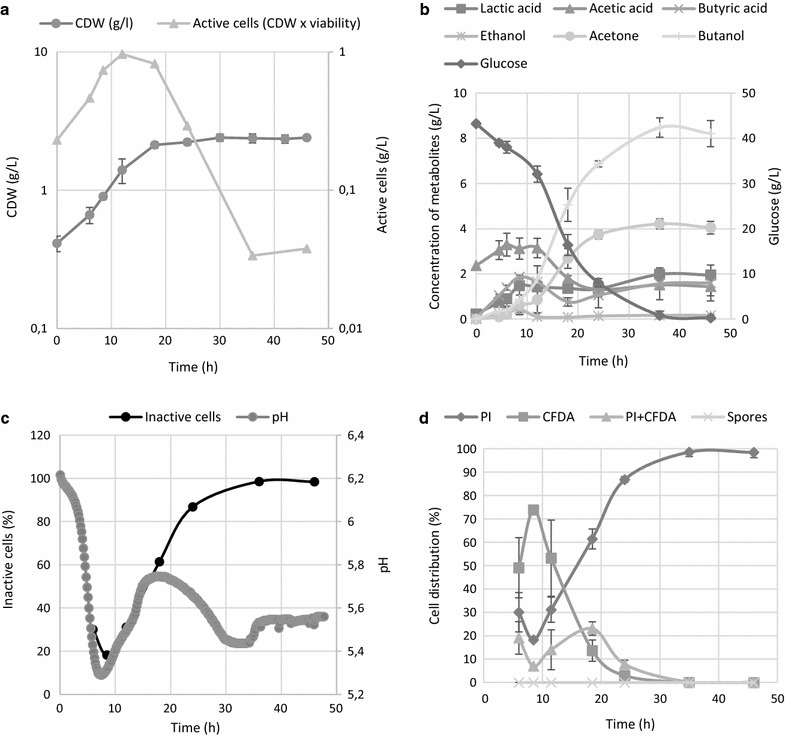
Time course of **a** cell growth and total amount of active cells (CDW multiplied by reciprocal value of inactive cells from chart **c**), **b** metabolite formation, **c** pH and percentage of inactive cells, **d** distribution of different sub-populations according to their LS and fluorescence staining patterns for the non-sporulating phenotype carried out on RCM medium. Error bars represent standard deviations of three independent biological replicates, and calculated values are presented without error bars

ABE fermentation carried out on RCM medium, where no spore formation was expected, showed quite similar production characteristics (Fig. 4) as the sporulating phenotype, with some exceptions. Low levels of solvents were formed from the start of the ABE fermentation, together with the production of organic acids. Lactic acid was formed in significantly higher titres and glucose was completely utilized. The first pH drop was comparable with previous cultivations in terms of time and pH but the following pH change was much slower, reaching the second maxima and minima later than was seen in the reference ABE fermentation (Fig. 1c). Although the sporulating phenotype is believed to be profitable for solvent formation, the maximum concentration of butanol produced on RCM medium was surprisingly high, reaching 8.5 g/L, even though no sporulation or typical cell thickening occurred. The sporulating phenotype produced 7.7 g/L of butanol (Fig. 1).

**Fig. 5:**
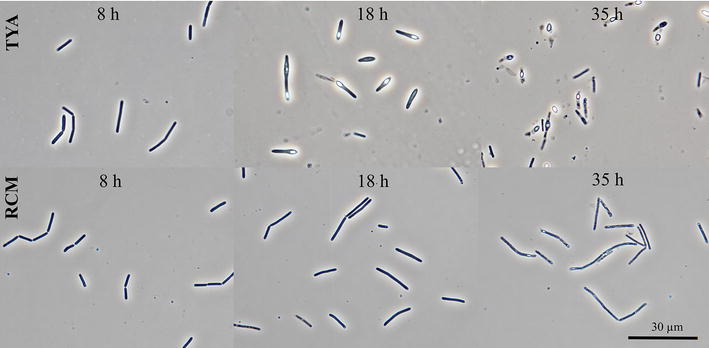
Morphology of the sporulating phenotype of *C. beijerinckii* grown on TYA and non-sporulating phenotype grown on RCM. Microphotographs were taken at the 8th, 18th and 35th hour during the mid-solventogenic phase

Even though apparent differences in morphologies and sporulation patterns were observed on TYA and RCM media, the curves representing changes in the total proportion of active cells in the population followed the same pattern, having their minima at the metabolic switch point. The 50% viability point corresponded to approx. the 16th hour of cultivation with a butanol concentration of less than 4 g/L, less than a half of the total butanol produced. No spores were formed, and this was confirmed microscopically (see Fig. 5) as well as by using FC (Fig. 4d). Similar to cultivation on TYA media, the proportion of solely CFDA stained cells declined over the cultivation period, starting from pH reversion and the total number of active cells peaked at the 18th hour.

### Growth on TYA medium supplemented with butanol

**Fig. 6:**
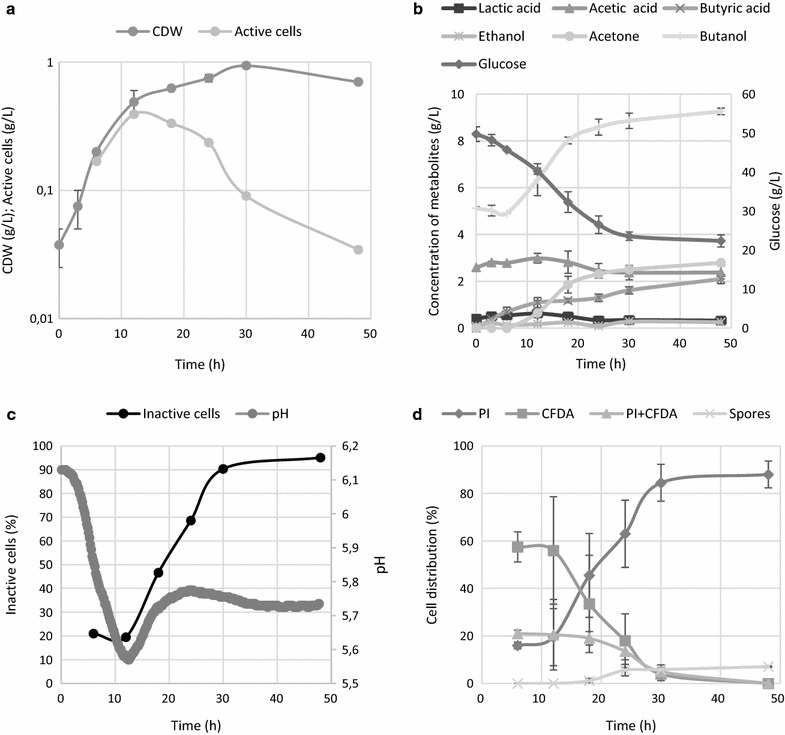
Time course of **a** cell growth and total amount of active cells (CDW multiplied by reciprocal value of inactive cells from chart **c**), **b** metabolite formation **c** pH and percentage of inactive cells, **d** distribution of different sub-populations according to their LS and fluorescence staining patterns for the sporulating phenotype and ABE fermentation carried out on TYA medium with external addition of 5 g/L butanol prior to fermentation. Error bars represent standard deviations of three independent biological replicates, and calculated values are presented without error bars

To determine the role of butanol in the early loss of viability during ABE fermentation, TYA medium (a typical sporulation life cycle was expected) was supplemented with butanol at a concentration 5 g/L prior the inoculation. The chosen concentration represented amount of butanol produced until the stage when the culture showed approximately 50% viability under sporulating conditions (Fig. 1). Cell growth was apparently slower with a significantly lower cell concentration and prolonged acidogenic phase that reached its minimal pH value approximately 5 h later than without a butanol challenge. Interestingly, profiles of production curves (Fig. 6) show that the onset of solvent formation occurred in the 6th hour of cultivation, similar to the first reference cultivation on TYA medium and was not accompanied by an increase in pH. The proportion of inactive cells revealed the same declining trend after the pH level recovered, even though it was about 5 h later than the start of solvent formation and corresponded to the rate of acid formation (pH trend) rather than solvent production, suggesting that pH plays an important role in the attenuation of viability during the clostridial life cycle. Furthermore, from the staining profile of the culture, where butanol stress was mimicked by the artificial addition of butanol, it was evident that the proportion of active cells in early stages of cultivation was as high as under conditions of no stress. This supports our assumption that *C. beijerinckii* NRRL B-598 is able to tolerate and adapt to sub-lethal concentrations of butanol and the presence of such butanol titres should not be responsible for the sharp decline in viability that was observed during previous ABE fermentation experiments. Moreover, the total butanol titre was the highest (9.3 g/L), although this was probably a consequence of lower acetone production. The sum of total ABE was 12.6 g/L, which is similar to both previous experiments, with 11.9 and 12.9 g/L ABE on TYA and RCM media, respectively.

### Decreased initial pH

The previous viability results were surprising taking into account the assumption that butyric acid accumulation together with a low pH are usually considered to be detrimental to cells and that solventogenesis serves as a rescue to overcome this unfavourable condition [1, 25]. Therefore, a decline in viability during the last stages of acidogenesis and a subsequent increase after the metabolic switch would be a logical outcome for this phenomenon. Such behaviour was observed when ABE fermentation started at a lower pH, pH 6.0, using TYA medium. Whereas a starting pH of 6.3 resulted in minimal values of pH 5.3, decreasing the starting pH to 6.0 resulted in lower minimum values being reached, around pH 4.9.

**Fig. 7:**
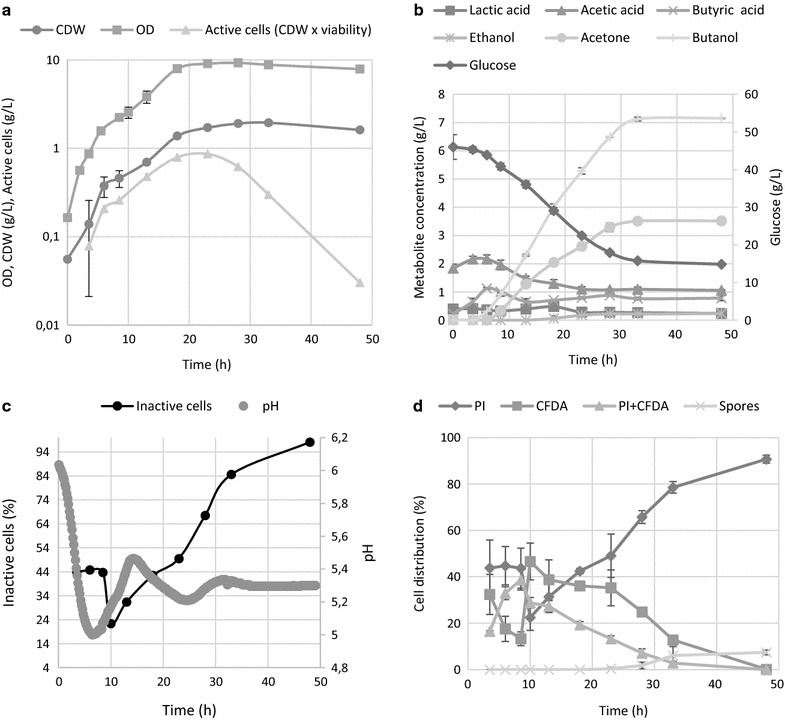
Time course of **a** cell growth and total amount of active cells (CDW multiplied by reciprocal value of inactive cells from chart **c**), **b** metabolite formation, **c** pH and percentage of inactive cells, **d** distribution of different sub-populations according to their LS and fluorescence staining patterns for the sporulating phenotype and ABE fermentation carried out on TYA medium with a lower initial pH value of 6.0. Error bars represent standard deviations of three independent biological replicates, and calculated values are presented without error bars

The comparison of pH and active cell curves in Fig. 7 clearly shows that the culture recovered shortly after the pH started to increase. In the acidogenic phase, the percentage of viable cells remained constant and was divided between solely or doubly stained cells where CFDA positive cells decreased at the expense of doubly stained ones. For a sample withdrawn at the 10th hour of cultivation, the number of solely CFDA positive cells increased sharply as a result of both culture revitalization and reduction of chain formation. After this apparent viability revival, FC data followed the same pattern as was observed in all previous experiments—cells proceed to death.

From the point of metabolite formation there was no apparent difference between cultivation carried out with an initial pH of 6.3 or 6.0 except that the lower starting pH provided lower titres of acids as well as solvents. The final spore number achieved was lower in comparison with the first cultivation (see Figs. 1 and 7) but otherwise the population exhibited a typical sporulating phenotype.

### Comparison of ABE yield and productivity

The main production characteristics were calculated according to formulas in “Methods” and are summarized in Table 1. The yield of butanol and solvents produced on glucose were the highest for fermentation carried out at a lower pH for a culture showing a typical, sporulation based, life cycle. When fermentations with a sporulation phenotype were compared, a lower initial pH led to decreased formation of acids and solvents together with decreased glucose consumption. The concentration of both butanol and total ABE was the highest on modified RCM, where no sporulation occurred, but the yield of solvents produced from glucose was lower. Contrary to the rest of experiments, all glucose was consumed until the end of the ABE process. A decreased yield of ABE for non-sporulating cells was in contradiction with the presumption that such a phenotype would be economically more advantageous as there would be no need for energy to be spent on sporulation.

**Table 1:** Butanol and solvent yields and productivity achieved under different cultivation conditions

	*Y*_B/G_	*Y*_ABE/G_	*P*_B_ (g L^−1^ h^−1^)	*P*_ABE_ (g L^−1^ h^−1^)	*Y*_B/CDW_	*Y*_ABE/CDW_
Sporulation phenotype TYA	0.22	0.34	0.23	0.35	3.08	4.76
Non-sporulation phenotype RCM	0.20	0.30	*0.25*	*0.37*	3.54	5.38
Butanol stress, TYA	0.15	0.27	0.12	0.21	*4.56*	*8.22*
Decreased initial pH, TYA	*0.23*	*0.35*	0.22	0.33	3.81	5.77

*Y*_B/G_ yield of butanol produced from consumed glucose (g/g)

*Y*_ABE/G_ yield of ABE produced from consumed glucose (g/g)

*P*_B_ (g L^−1^ h^−1^)—volumetric productivity of butanol

*P*_ABE_ (g L^−1^ h^−1^)—volumetric productivity of ABE

*Y*_B/CDW_—amount of butanol produced per unit of dry weight biomass (g/g)

*Y*_ABE/CDW_—amount of ABE produced per unit of dry weight biomass (g/g)

The highest values of all parameters are given in italics

Cells subjected to butanol stress had the lowest volumetric productivities and yields related to glucose consumption, but simultaneously produced more butanol and ABE in relation to biomass concentration; this yield reached 8.2 g solvent per 1 g of dry biomass, adaptation mechanisms and stress cell response might play a role in this phenomenon where more energy for cell maintenance was needed, and production was prioritized to growth.

## Discussion

High butanol toxicity is often presented as a key bottleneck hindering the achievement of higher solvent titres during ABE fermentation. The complete range of cell stress responses has been described in detail, but the exact mechanism of tolerance and its relationship to metabolism has not been fully elucidated [33]. Engineering of strains for increased butanol tolerance has been successful many times and some studies have shown that enhanced tolerance can also improve production [45–47]. On the other hand, there is an evidence that higher tolerance does not necessarily increase production even though tolerance is significantly increased (e.g. [30]). This could be explained by the existence of additional regulatory mechanisms involved in solvent production. Viability data obtained in this work clearly show a decrease in culture viability in relation to the pH breakpoint rather than the concentration of butanol, supporting the assumption that a tolerance-independent/(or of unknown dependence) regulatory mechanism is involved in this phenomenon. A decisive point for such a mechanism could be the onset of sporulation—an attempt by cells to survive, giving preference to the development of a dormant status while simultaneously sacrificing vegetative cells in favour of the next generation. Spontaneous large scale autolysis after the exponential growth phase can be observed and is attributed to a potential need for nutrients required for sporulation [34]. This theory is supported by our observation that cell dry weight (CDW) in later stages declined only in the sporulating phenotype, but this process takes place far later than when viability starts to decline and thus autolysis cannot be an explanation for the gradual loss in viability. Rather, it might be associated with a strategy for long term survival of the population, which has been developed in nature by some bacterial populations and which is coupled with alternating high and low cell densities and re-cycling/reutilization of material originating from dead cells [48]. Allcock et al. [49] measured the autolytic activity of *C. acetobutylicum* P262 and found that it peaked in the middle of exponential phase; however, autolysins are enzymes with a wide range of physiological functions and their increased activity in exponential phase is more probably related to an increased need for peptidoglycan turnover and cell enlargement rather than digestion of metabolically inactive cells. Massive autolysis of commercial strains was known to occur during industrial processes and this was assumed to be due to higher concentrations of butanol. Van Der Westhiuzen et al. [50] described the increased tendency to autolysis at butanol concentrations of 7–16 g/L whereas higher butanol concentrations had the opposite effect and inhibited autolysis. A comparison of growth characteristics obtained for sporulating and non-sporulating phenotypes of *C. beijerinckii* NRRL-B598 shows that only for the sporulating phenotype was there a decrease in OD as well as CDW after reaching their maximum. CDW of biomass grown on modified RCM with no spore formation remained constant until the end of the experiment (zero viability)—no apparent autolysis was observed. Viability development during these two ABE fermentations seemed to be very similar, which leads us to the assumption that a natural decline in viability at early stages of fermentation is not influenced by the rate of autolysin formation or sporulation.

The solventogenic phase of ABE fermentation is generally associated with a cessation of cell growth and cell metabolic activity is maintained until the accumulation of solvents reaches an inhibitory level [1]. However, cessation or slowing of growth is not a prerequisite for solventogenesis for all strains across the solventogenic clostridia. This feature is strain dependent; e.g. *C. pasteurianum* ATCC 6013 produced butanol from glycerol simultaneously with cell growth [51] in contrast with *C. acetobutylicum* ATCC 824, where solvent production was connected with stationary phase [37, 38, 52]. *C. beijerinckii* NRRL B-598 used in this study reduced growth rate shortly before the pH breakpoint but continued in growth after commencement of solventogenesis. Similar growth characteristics can be found for *C. beijerinckii* NCIMB 8052 (e.g. [53]). Based on phylogenetic analysis [54], *C. beijerinckii* NRRL B-598 shares high genome homology with *C. beijerinckii* NCIMB 8052 and therefore similar behaviour seems probable. Jones and Woods [1] mention the fact that even though growth is stopped, CDW and OD values can still increase due to the accumulation of granulose and changes in cell morphology; however, this was not the case for *C. beijerinckii* NRRL B-598, where the OD_600_ for reference cultivation on TYA medium increased more than 5 times since the change in growth rate. Moreover, in selected samples, cell concentration was measured by FC and a good correlation between OD and cell number was obtained (data not shown). Nevertheless, all such data assessing a bulk culture do not possess a deeper insight into changes of cell physiology and culture heterogeneity. The FC approach used in this study produced valuable information about the state of individual cells beyond culturability, where only vegetative cells and early sporulation states (reversible) can be quantified without additional treatment. Even though FC can offer a nearly infinite number of options for analysis of particular cell features at the individual cell level (see, e.g. [55, 56]), its application in the analysis of microbial cells is difficult due to the small size of cells, enabling only a limited number of probes to be applied at the same time (generally only two for bacterial cells) and the enormous variability in their metabolism and structure causes problems in setting up reliable assays [57]. This applies doubly for solventogenic clostridia, which have long resisted efforts to identify and implement a protocol to determine their viability and physiological state based on fluorescence staining [36–38, 58]. Nevertheless, these studies provided a robust basis for the design of quick and reliable methodologies that were applied for *C. beijerinckii* [35], *Clostridium pasteurianum* [11] and *Clostridium tetanomorphum* [59]. From our results, as well as those published, it is clear that viability was seldom close to 100%, as can be observed for a wide range of microorganisms in exponential phase [60–62]. Generally, at least four populations can be distinguished when multi-parameter cytometry and double fluorescence staining is applied [42, 62] to a bacterial community. *C. beijerinckii* was not an exception and four clearly recognisable populations were apparent on FC dot-plot diagrams. The development of a staining pattern clearly indicated the beginning of a decline in viability together with the pH breakpoint and onset of solventogenesis, which was consistent with previous observations for *C. beijerinckii* CCM 6218 [59] and *C. beijerinckii* NRRL B-598 [35].

As pH has an important role in the rearrangement of metabolic pathways [63], it might also be a key factor in the decision to attenuate viability. The butanol concentration at the decisive point was too low to be responsible for this change although it certainly contributed to the later decline in viability as hydrophobic solvents can alter membrane fluidity, subsequently leading to destabilization of membrane-bound complexes, and have a negative impact on their functions. Other solvent effects are biomolecular misfolding, damage, and generation of reactive oxygen species (ROS) [64, 65]. A combined effect of carboxylic acids and solvents should be considered as they both act primarily on the cell membrane and influence overall energy balance. Decreased extracellular pH requires more ATP for H+ ATPase to maintain a constant internal pH and at the same time solventogenesis provides less ATP than acidogenesis. Such a combination can cause a collapse of cellular functions and energy dissipation. However, Wang et al. [66] showed that low butanol titres (up to 0.8%) had little effect on ΔpH maintenance, but low pH and the presence of carboxylic acids might still enhance butanol toxicity. All of these factors can result in cell death, although solvent concentration at the final stage of ABE fermentation was considerably lower for *C. beijerinckii* NRRL B-598 (around 8 g/L butanol and 12 g/L ABE) compared to related clostridia (up to approx. 20 g/L [67]).

Nevertheless, we hypothesise that there might be some additional mechanisms involved in cell culture inhibition, activated at some point of the metabolic switch and independent of sporulation or the actual solvent concentration when present at low titres. This is consistent with the observations of Grimmler et al. [68] that in *C. acetobutylicum,* transcriptional regulation of genes involved in the stress response are linked to the metabolic shift and not butanol stress.

Since a range of genes was found to be responsible for solvent tolerance and/or increased production, we think that one of the factors playing an important role in regulating the relationship between cell death and solvent concentration might be Spo0A (the supposed master regulator of solventogenesis and sporulation). This is supported by Kolek et al. [44], who overexpressed *Spo0A* in *C. beijerinckii* NRRL B-598 and came to the conclusion that increased transcription of *Spo0A* led to a cessation of production and metabolism at a low ABE concentration. The same was observed by Harris et al. [69], whose *Spo0A* transformed *C. acetobutylicum* strain produced higher titres of solvents than the control strain but lower than that of parental. Reversely, Sandoval et al. [51]. inactivated *Spo0A* in *C. pasteurianum* and reached higher production characteristics. Thus Spo0A, or another factor, might be jointly responsible for the decline in viability. Such a mechanism should ensure culture survival even in a dormant form. Recently, it was proposed for *C. acetobutylicum* ATCC 824 [22, 70] that cell density might be the factor that could cause a pleiotropic response in the culture under the same conditions. Xue et al. [22, 70] considered a quorum sensing mechanism, together with AbrB regulators, to be of key importance when a culture of a specific cell density reached a decisive point defined, e.g. by a specific pH and/or a specific butanol concentration. This might also be the case for our strain; however, this issue needs a more in-depth investigation. *C. beijerinckii* strains, including strain NRRL B-598, generally contain genes for the *agr*-based quorum sensing system [4], which was found to be responsible for regulation of sporulation in *C. acetobutylicum.* At the same time, the *agr*-based system in *C. acetobutylicum* did not significantly influence solventogenesis and final solvents titres [71, 72].

Jones and Woods [1] presented industrial fermentation as a process where full maturation of spores was not usually reached due to accumulation of toxic butanol concentrations, so strains with a disrupted mechanism of self-preservation might have been selected for industrial applications. Whereas the clostridial response to butanol challenge is complex and the regulation of tolerance and production remains a scientific challenge, adaptive laboratory evolution and culture domestication [20] can lead to the development of promising butanol producers. Irrespective of whether the strains are prepared by targeted manipulations or naturally selected, FC-based analysis can be a valuable tool for the rapid selection of the best high producing and tolerant candidates [25].

Next to butanol and ABE, a low pH, together with the influence of organic acids (the toxicity of which is pH dependent [73]) are another lethal combination from which, solventogenesis together with acid reutilization should be the rescue action. This is clearly visible from the experimental data acquired under lower initial pH, where the pH breakpoint took place at values slightly under pH 5. Growth under this acidic condition negatively influenced the proportion of metabolically active cells in the culture at the first stages of cultivation and considerably improved after the pH increased. Following that, the viability profile followed the same trend as in all previous experiments. This demonstrates the assumption regarding directed and inevitable cell fate in batch culture.

Simultaneously with screening for cell viability during ABE fermentation, ABE yield, volumetric productivity and specific production were compared. Cells challenged by butanol revealed low volumetric productivity but superior solvent yield relative to biomass, which was significantly decreased comparing to the reference cultivation. A lower biomass concentration at butanol challenge was already observed (e.g. [74]) and the results are in agreement with the previously described inverse proportion of specific and volumetric productivity [75]. As the viability of the culture with added butanol did not deviate from the patterns of other experiments, a necessarily lower total number of cells had to produce similar amounts of butanol. Another interesting output was the lower yield of ABE from glucose, as found for the non-sporulating phenotype, because sporulation indisputably represents a demand for additional energy and therefore should be unfavourable for economic aspects of commercial production [76]. The non-sporulating phenotype used in this work was induced by cultivation conditions, which were discovered by chance and, unfortunately, a deeper understanding of regulatory mechanisms or triggers of a particular phenotype are still under investigation.

Another interesting question associated with the FC analyses of different populations is which part of the population is responsible for solvent production. In the past [1] it was generally considered, at least for *C. acetobutylicum,* that solvents were formed by sporulating, granulose-containing cells (clostridium-like cells). However, this was contradicted by multiple observations of solvent producing asporogenous cultures [68, 77] and others. In addition, Tracy et al. [37], based on FC analysis, hypothesized that a proportion of vegetative cells within the clostridial population was actually responsible for solvent production. Our results confirm this observation and support the hypothesis.

## Conclusion

Viability changes of *C. beijerinckii* NRRL B-598 during ABE fermentations and their relationship to production characteristics and pH changes have unambiguously proven that an increase in the proportion of inactive cells (those stained by PI) was not only dependent on solvent or acid concentrations but was also connected to metabolic transitions. The most important point in the decision regarding cell destiny seemed to be tightly interconnected with the pH breakpoint and the onset of solventogenesis. Moreover, it was shown that PI and CFDA could be used for a physiological heterogeneity assay of solventogenic clostridia, and together with multi-parameter flow cytometry, could be an invaluable tool in these studies.

## Methods

### Microorganism

All the experiments were carried with *C. beijerinckii* NRRL B-598 obtained from NRRL/ARS Culture Collection, Peoria, Illinois, USA as *C. pasteurianum* NRRL B-598. Based on its genome information [78] it was proposed for re-classification to *C. beijerinckii* species [54]. Cells were maintained in a form of spore suspension kept in distilled water at a temperature of 4 °C. To initiate spore germination prior to cultivation, spore suspensions were subjected to 2 min treatment at 80 °C.

### Fermentation experiments

*Clostridium beijerinckii* NRRL B-598 was grown in parallel Multiforce bioreactors (Infors HT, Bottmingen, Switzerland) equipped with an electrode for on-line pH measurements. Conditions were as follows: 37 °C, agitation 200 RPM, initial pH 6.3 (experiments with lower initial pH started with pH 6.0; 10% NaOH solution was used for pH regulation prior to cultivation, if necessary). Anaerobic conditions were established with CO_2_ or N_2_ and maintained using an inflated bag connected to a bioreactor fitting that terminated above the liquid level. Medium (630 mL) was inoculated with 70 ml of overnight culture prepared in respective culture broths with 20 g/L of glucose in the Concept 400 (Ruskinn, UK) anaerobic chamber. Composition of the TYA medium for bioreactor experiments was: 50 g/L glucose, 2 g/L yeast extract (Merck, Darmstadt, Germany), 6 g/L tryptone (Sigma-Aldrich, St. Louis, Missouri, USA), 0.5 g/L KH_2_PO_4_, 3 g/L ammonium acetate, 0.3 g/L MgSO_4_·7H_2_O and 0.01 g/L FeSO_4_. Modified RCM broth contained: 50 g/L glucose, 10 g/L tryptone (Sigma-Aldrich, St. Louis, Missouri, USA), 10 g/L meat extract (Merck, Darmstadt, Germany), 3 g/L yeast extract (Merck, Darmstadt, Germany), 5 g/L sodium chloride and 3 g/L sodium acetate.

### Cell staining procedure, flow cytometry, microscopy

Cells were harvested from the bioreactor through a sampling valve with the help of self-generated overpressure to prevent oxygen exposure. Cells were immediately washed twice with sterile physiological saline solution (1 min, 3000×*g*) and diluted to OD 0.5 ± 0.1 (measured at 600 nm, path length 1 cm). Propidium iodide stock solution (2 μL) (PI, 1 mg/mL) and 2 µL of 6-carboxy-fluorescein diacetate (CFDA, 1 mg/mL) were added to 200 µL of prepared aliquots of cell suspension. Both fluorescent probes were purchased from Sigma-Aldrich (St. Louis, Missouri, USA). Samples were mixed thoroughly and incubated in the dark at room temperature. After 7 min of incubation, 50 µL of cell suspension was mixed with 2 mL of freshly filtered (0.22 µm) demineralised water and immediately measured in a BD Accuri C6 (BD Accuri Cytometer Inc., USA) flow cytometer. The FC was equipped with 20 mW, 488 nm, Solid State Blue Laser. For each sample, at least 10,000 particles were analysed and data were processed as shown in the Fig. 2. Forward scatter (FSC) and side scatter (SSC) signals were used to trigger and define the cell population. Green (FL1; 515–565 nm) and red (FL3; > 605 nm) fluorescence emission was recorded and the FL3 signal was compensated with 3% of FL1. The dot-plot diagram was divided into four quadrants according to the staining pattern. Those particles that were in a non-stained region (lower left) were subsequently analysed for their FSC and SSC parameters and particles with uniform and typical light scatter signals (LS) were counted as spores and their ratio was calculated to a population gated in the first step. For microscopic examination of *C. beijerinckii* morphology and staining pattern, phase contrast and epi-fluorescence microscopy were employed using an Olympus BX51 microscope (Olympus, Tokyo, Japan) equipped with a 120 W mercury vapour arc lamp and a U-MWB2 filter cube (excitation 460–490 nm, emission + 520 nm). The microphotographs in this article are real images shot directly by camera (EOS 600D, Cannon, Tokyo, Japan). To illustrate development of the staining profile during sporulation (Fig. 3), particular states were chosen from different images and combined into one image.

### Analysis of cell growth and metabolite formation

Cell growth was measured as cell dry weight (CDW) after washing and drying to constant weight at 105 °C and/or as the OD_600_ of the culture (Varian Cary 50 UV–VIS spectrophotometer, Varian Inc.). Metabolite and glucose concentrations were analysed by HPLC (Agilent series 1200, Agilent, Spain) under the following conditions: mobile phase 5 mM H_2_SO_4_, flow rate 0.5 mL/min, column temperature 60 °C, injection volume 20 µL, stationary phase IEX H polymer (Watrex, Czech Republic), refractive index detection.

#### Formulas

*Y*_B/G_ yield of butanol produced from consumed glucose (g/g)

$$Y_{{{\text{B}}/{\text{G}}}} = \frac{{c_{\text{Bmax}} - c_{\text{Bt0}} }}{{c_{\text{Gt0}} - c_{\text{Gfin}} }}$$

*Y*_ABE/G_ yield of ABE produced from consumed glucose (g/g)

$$Y_{{{\text{ABE}}/{\text{G}}}} = \frac{{c_{\text{ABEmax}} - c_{\text{ABEt0}} }}{{c_{\text{Gt0}} - c_{\text{Gfin}} }}$$

*P*_B_ (g L^−1^ h^−1^)—volumetric productivity of butanol

$$P_{\text{B}} = \frac{{c_{\text{Bt33}} - c_{\text{Bt0}} }}{{t_{33} }}$$

*P*_ABE_ (g L^−1^ h^−1^)—volumetric productivity of ABE

$$P_{\text{ABE}} = \frac{{c_{\text{ABEt33}} - c_{\text{ABEt0}} }}{{t_{33} }}$$

*Y*_B/CDW_—amount of butanol produced per unit of dry weight biomass (g/g)

$$Y_{{{\text{B}}/{\text{G}}}} = \frac{{c_{\text{Bmax}} - c_{\text{Bt0}} }}{{c_{\text{CDWmax}} }}$$

*Y*_ABE/CDW_—amount of ABE produced per unit of dry weight biomass (g/g)

$$Y_{{{\text{ABE}}/{\text{G}}}} = \frac{{c_{\text{ABEmax}} - c_{\text{ABEt0}} }}{{c_{\text{CDWmax}} }}$$

*c*_Bmax_, *c*_ABEmax_, *c*_CDWmax_, the highest achieved concentration of the respective parameters, butanol, ABE and CDW in g/L.

*c*_Bt0_, *c*_ABEt0_, *c*_Gt0_—concentration of butanol, ABE and glucose after bioreactor inoculation at time 0 h in g/L.

*c*_Gfin_—concentration of glucose (g/L) at the end of experiment

*c*_Bt33_, *c*_ABEt33_, concentration of respective substances after 33 h (period when most of the processes were finished in all experiments). In case sampling was not done exactly at this time, a linear interpolation was used to calculate the values.

*t*_33_—time corresponding to 33 h of cultivation.

## Abbreviations

ABE: acetone–butanol–ethanol
*agr*: accessory gene regulator
CDW: cell dry weight
CFDA: carboxyfluorescein diacetate
FC: flow cytometry
FSC: forward scatter
LS: light scatter
PI: propidium iodide
RCM: reinforced clostridial medium
SSC: side scatter
TYA: tryptone-yeast extract-acetate medium
